# A Rare Case of Polymicrobial Empyema Associated With COVID-19 Infection Complicated by a Bronchopleural Fistula

**DOI:** 10.7759/cureus.86024

**Published:** 2025-06-14

**Authors:** Dhiraj R Regmi, Sandeep Kshetri, Laxman Wagle, Sabita Regmi

**Affiliations:** 1 Internal Medicine, Nepalese Army Institute of Health Sciences, Kathmandu, NPL; 2 Internal Medicine, MedStar Franklin Square Medical Center, Baltimore, USA; 3 Internal Medicine, Ascension Saint Agnes Hospital, Baltimore, USA; 4 Internal Medicine, Tribhuvan University Institute of Medicine, Kathmandu, NPL

**Keywords:** antibiotics, bacterial superinfection, bronchopleural fistula, covid-19, covid pneumonia, empyema, pleural fistula, polymicrobial, thoracostomy

## Abstract

Empyema is a rare but serious complication in patients with COVID-19, and its association with bronchopleural fistula (BPF) is even more uncommon. We present the case of a 40-year-old woman with a history of intravenous drug use who developed polymicrobial empyema in the setting of COVID-19, further complicated by BPF. She presented with hypoxic respiratory failure, leukocytosis, and lactic acidosis. Imaging revealed a large right-sided hydropneumothorax and bilateral lung opacities. Broad-spectrum antibiotics, antivirals, and corticosteroids were initiated, and 3 liters of purulent fluid were drained via chest tube, confirming empyema. Cultures identified multiple pathogens, including *Bacteroides fragilis*, *Arcanobacterium hemolyticum*, methicillin-sensitive *Staphylococcus aureus*, and Group C/G streptococci. Persistent air leak and incomplete lung re-expansion led to a diagnosis of BPF. The patient was successfully managed with prolonged antibiotic therapy and chest tube drainage without requiring surgical or bronchoscopic intervention. This case highlights the importance of recognizing and conservatively managing small BPFs in COVID-19-associated empyema, emphasizing the potential for non-surgical resolution in select cases.

## Introduction

Bacterial pneumonia in the context of COVID-19 is common, often caused by organisms such as methicillin-sensitive *Staphylococcus aureus* (MSSA) and *Pseudomonas aeruginosa* [1]. Empyema refers to active suppuration within the pleural space. Empyema is confirmed by one out of three criteria: pleural fluid culture or Gram stain showing organisms, presence of grossly purulent fluid on thoracentesis or thoracotomy, or biochemical evidence as indicated by pleural fluid with a pH <7.2 and glucose <40 mg/dL. COVID-19 sets the stage for empyema by causing thrombosis of interstitial blood vessels, parenchymal necrosis, and later small pneumothoraces. Other mechanisms like corticosteroid use, rupture of pneumatoceles with bacterial co-infection, and the presence of alveolo-pleural fistulas are also implicated to a lesser degree [2]. Empyema in patients with COVID-19 is rare, with only 43 cases reported in the literature, and is associated with a mortality rate of approximately 23%. Bronchopleural fistula (BPF), which is defined as a direct communication between the bronchus and pleural cavity, was documented in five of these 43 cases [3]. We present a unique case of empyema in an intravenous drug user with COVID-19, complicated by the development of a BPF.

## Case presentation

A 40-year-old woman with a history of substance use disorder presented with dyspnea and pleuritic chest pain. She was noted to be tachycardic and tachypneic with normal blood pressure. She was placed on a non-rebreather mask for hypoxia and later transitioned to BiPAP for persistent acute hypoxic respiratory failure.

**Figure 1:**
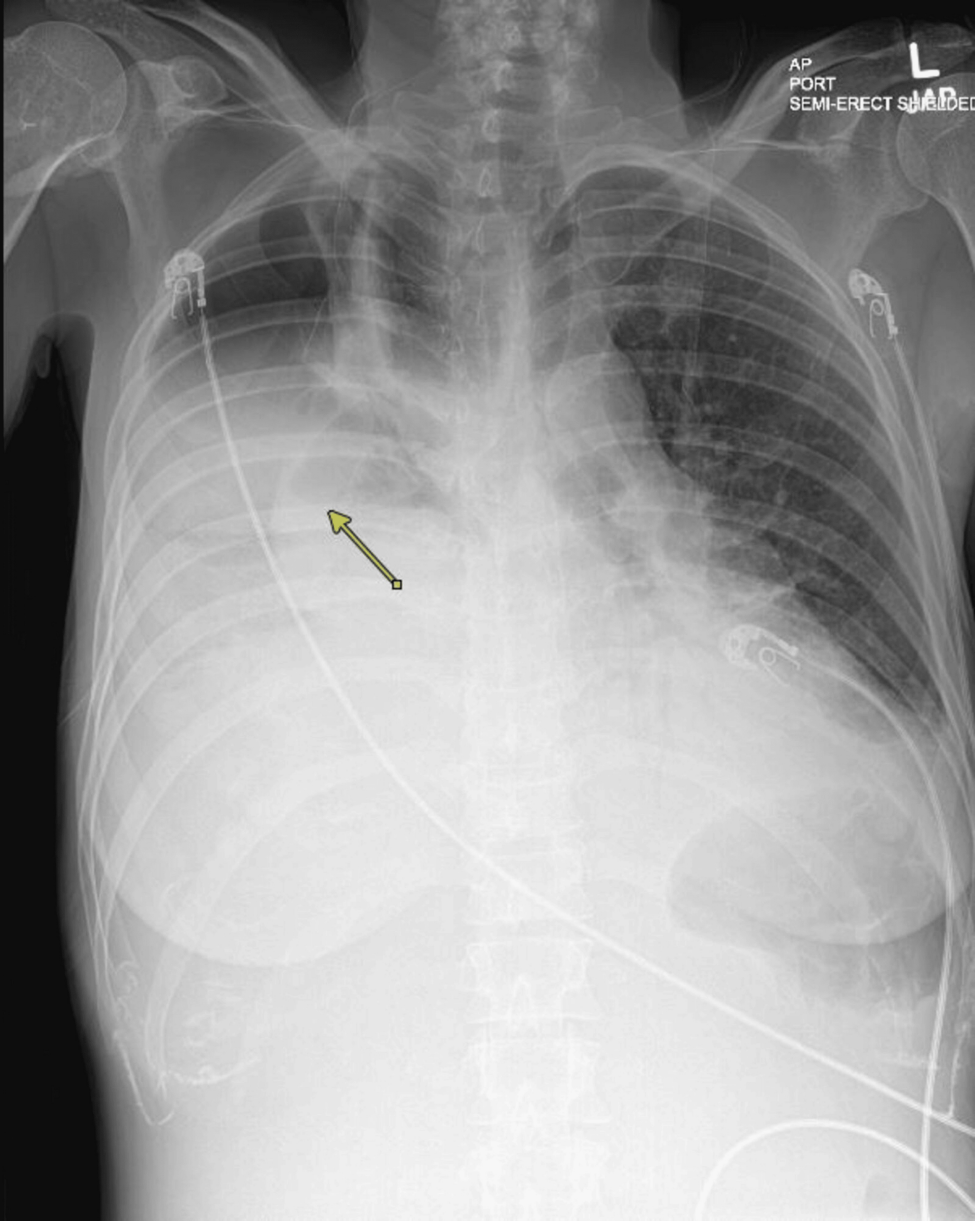
Initial chest X-ray on presentation ​​demonstrating a large right hydropneumothorax (marked with arrow) and left retrocardiac consolidation concerning pneumonia

**Figure 2:**
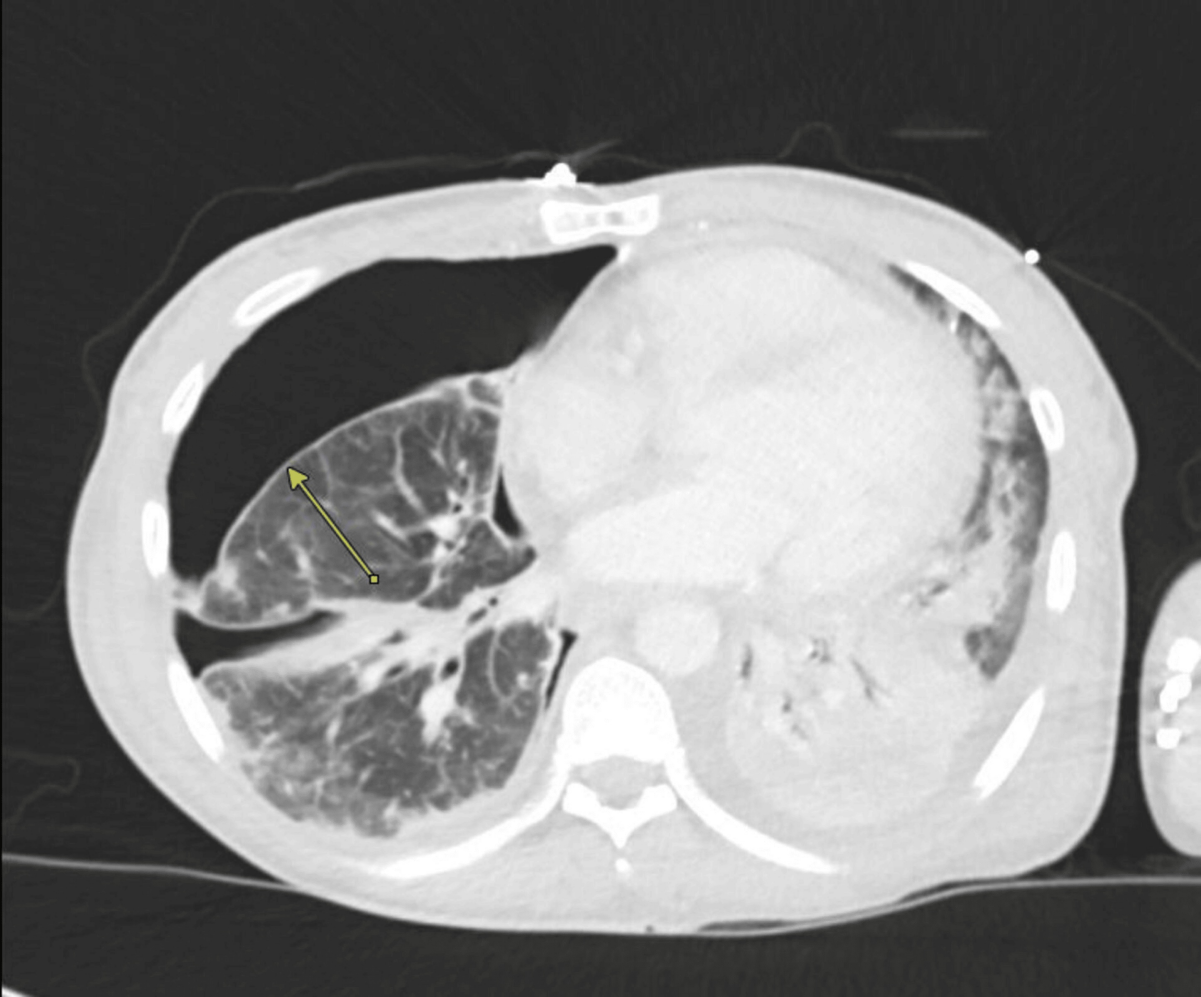
Chest CT with contrast on Day 3 showed decreased fluid component but a stable large amount of air within the right hydropneumothorax (arrow)

Physical exam was significant for bilateral rhonchi with decreased breath sounds on the right side and chronic wounds in bilateral lower extremities. Labs revealed leukocytosis and lactic acidosis (Table 1). COVID-19 RNA PCR was positive. Chest X-ray showed large right hydropneumothorax and left retrocardiac consolidation (Figure 1). CT angiography chest showed massive right hydropneumothorax with loculation, consolidation of the right lung with multiple parenchymal air bronchograms, and multifocal ground glass with confluent opacities throughout the left lung, suggestive of post-viral bacterial pneumonia (Figure 2). The patient was started on broad-spectrum antibiotics, vancomycin and piperacillin-tazobactam, as well as remdesivir and dexamethasone. She had a right-sided pigtail catheter placed, draining 3 liters of purulent fluid. Pleural fluid analysis was consistent with empyema (Table 1). 

**Table 1:** Initial laboratory values Units: mEq/L, milliequivalents per liter; mg/dLm milligrams per deciliter; U/L, units per liter; /μL, per microliter; /mm³, per cubic millimeter Abbreviations: BUN, blood urea nitrogen; AST, aspartate aminotransferase; ALT, alanine aminotransferase; LDH, lactate dehydrogenase; WBC, white blood cell count; RBC, red blood cell count

Test	Result	Reference/normal range
Serum electrolytes and metabolites		
Sodium	134 mEq/L	135–145 mEq/L
Potassium	2.8 mEq/L	3.5–5.0 mEq/L
Chloride	98 mEq/L	98–106 mEq/L
Bicarbonate	25 mEq/L	22–29 mEq/L
BUN	14 mg/dL	7–20 mg/dL
Creatinine	0.42 mg/dL	0.6–1.3 mg/dL
Glucose	158 mg/dL	70–200 mg/dL
Calcium	8.2 mg/dL	8.5–10.5 mg/dL
Magnesium	1.9 mEq/L	1.7–2.2 mEq/L
Liver function tests, lactic acid, and LDH		
Total bilirubin	1.2 mg/dL	0.1–1.2 mg/dL
Direct bilirubin	0.87 mg/dL	0–0.3 mg/dL
AST	28 U/L	10–40 U/L
ALT	10 U/L	7–56 U/L
Alkaline phosphatase	246 U/L	44–147 U/L
Albumin	3.1 g/dL	3.5–5.0 g/dL
LDH	369 U/L	140–280 U/L
Lactic acid	4 mmol/L	0.5–2.2 mmol/L
Complete blood count		
WBC	14,670 /μL	4,000–11,000 /μL
Hemoglobin	9 g/dL	13.5–17.5 g/dL (M), 12–16 g/dL (F)
Platelets	603,000 /μL	150,000–450,000 /μL
Pleural fluid analysis		
Appearance	Turbid	Clear to straw-coloured
RBCs	66,000 /mm³	<5,000 /mm³
WBCs	142,100 /mm³	<1,000 /mm³
Glucose	4 mg/dL	>60 mg/dL
LDH	>4,500 U/L	<200 U/L
pH	6.1	>7.2

**Figure 3:**
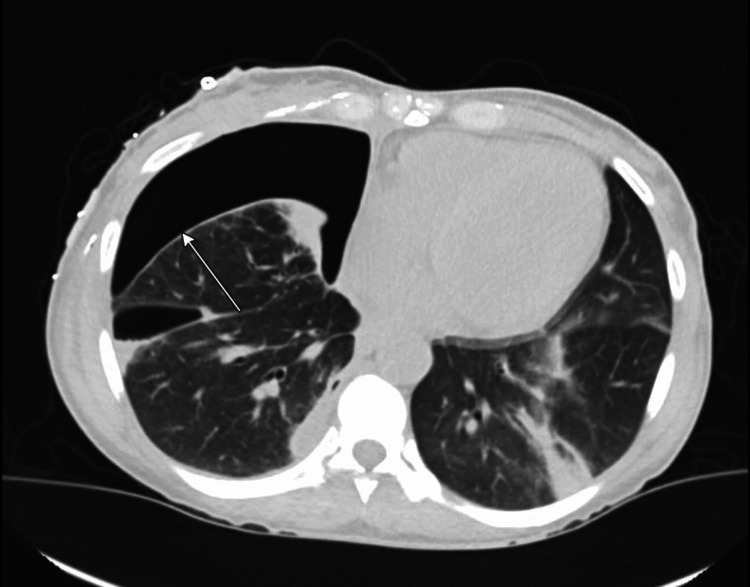
Chest CT on Day 9 showed persistent right-sided large pneumothorax (arrow) despite an indwelling thoracotomy tube and improving consolidation

The blood culture grew *Bacteroides fragilis*, *Arcanobacterium hemolyticum*, and MSSA. Pleural fluid culture grew Group C and G beta-hemolytic streptococci and *Arcanobacterium hemolyticum*. Antibiotics were narrowed to cefazolin and metronidazole. Subsequent blood cultures were negative. She continued to have purulent discharge via the intrapleural pigtail catheter. Her chest CT scan on Day 3 showed incomplete re-expansion of her right lung (Figure 3). On Day 4, a second right intrapleural pigtail catheter was placed in a more superior position. Despite this, pneumothorax persisted until Day 11 even after the consolidation was stable. However, the patient was able to saturate well in the room air. Due to ongoing air leak through the water seal drainage, incomplete lung re-expansion, and persistent pneumothorax, a diagnosis of BPF with trapped lung was made. This is a condition where the lung cannot fully re-expand due to a fibrous peel on the visceral pleura, which mechanically restricts the lung even after the pleural space has been drained. Other diagnoses that were considered were alveolo-pleural fistula and persistent pneumothorax, but were ruled out because of the clinical picture.

The lower chest tube was removed eventually on Day 12 after improvement in air leak. The patient was discharged with her right intrapleural pigtail catheter connected to a Heimlich valve, a one-way flutter valve used to drain air or fluid from the pleural space without allowing air to re-enter. She was discharged with close pulmonology and thoracic surgery follow-up. Intravenous antibiotic therapy was continued as an outpatient for four weeks.

**Figure 4:**
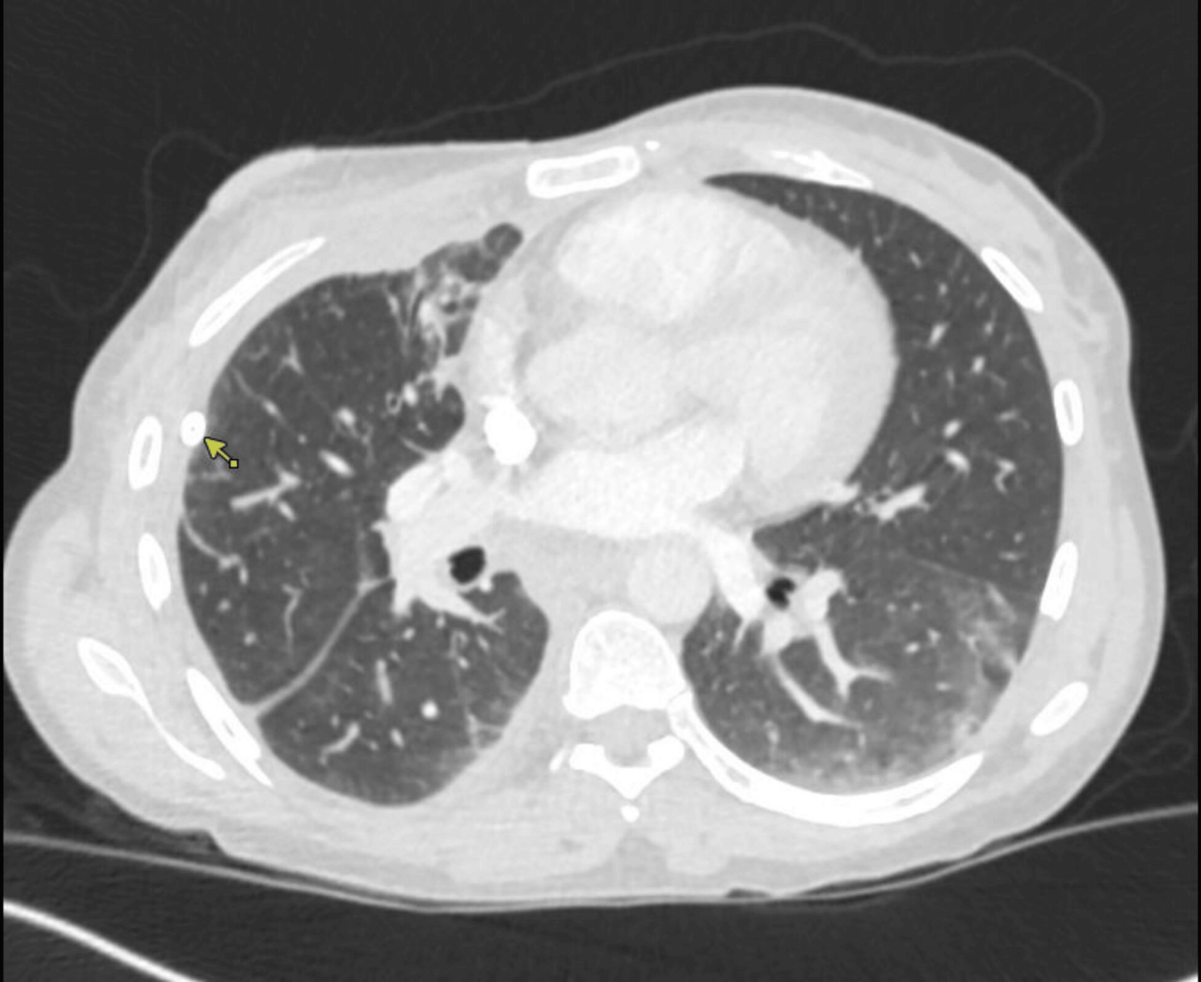
Chest CT on Day 40 showing the right-sided chest tube in place (arrow), patchy, right base rounded atelectasis and left base atelectasis

A follow-up chest CT on Day 40 showed the presence of a chest tube with patchy atelectasis (Figure 4). The chest tube was removed later.

## Discussion

BPF is a rare complication, usually secondary but not limited to lung resection surgery. It is defined as an abnormal communication between the pleural space and the airway. BPFs can occur secondary to a multitude of factors. In a study conducted by Puskas et al., it was found that the most frequently associated factor with BPFs was right pneumonectomy, followed by pneumonia, radiation therapy, prolonged mechanical ventilation, recurrent carcinoma, and tuberculosis [4,5]. Infectious etiology with anatomical aberrations like blebs further increases the risks for BPFs. 

Secondary infections leading to necrotizing pneumonia in COVID-19 can result in spontaneous pneumothorax as well as empyema and BPFs, causing a delay in diagnosis. Empyema carries a high risk of one-year mortality, ranging from 18.8% to 32.3% [3]. Empyema with BPFs, however, carries a poor prognosis resulting from percolation of empyema and perpetuation of infection. Physiological tension pneumothorax secondary to BPFs can cause acute decompensation requiring more interventions. This results in a long and complicated clinical course with mortality up to 67% [6]. Prompt identification and management of BPF is, therefore, of paramount importance [7-9]. 

BPF evaluation comprises clinical, imaging, and bronchoscopic assessments that confirm air leakage from a significant lobar or segmental bronchus to the pleural space. Clinically, persistent pleural air leak along with a change in the appearance of pre-existing pleural air fluid level are subtle signs suggestive of BPFs [8,10]. Diagnostic tools include chest computed tomography (CT) scans and flexible bronchoscopy, which typically reveal signs of BPF with fluid-air collections. In clinical practice, conventional modalities often fail to show smaller fistulas, necessitating multiple invasive procedures [8]. Newer modalities like multidetector computed tomography (MDCT) and advanced post-processing tools (virtual bronchoscopy) can help identify and localize the BPFs accurately and provide relevant additional information for surgical planning. MDCT should be considered the initial and primary diagnostic modality for the clinically suspected BPF instead of conventional CT. But this use case of MDCT may be limited because of its availability, in which case, conventional CT, along with subtle clinical signs, can help in prompt diagnosis of BPFs, especially a small BPF that may go unnoticed [9-11].

If the BPF is associated with a pleural infection, as in our case, prompt drainage and washout of the hemithorax while protecting the contralateral lung is warranted. Providers must be cautious of factors like pulmonary flooding, endobronchial contamination, and tension pneumothorax that can potentially cause acute worsening of the patient. Patients who experience profound hypoxia and deteriorating clinical condition in the background of COVID-19 or BPF-related complications can be managed with mechanical ventilation with unique settings or ECMO. After initial stabilization of patients, early initiation of broad-spectrum antibiotics and optimum nutritional rehabilitation will play a very important role in healing and quick recovery. Definitive repair should be delayed until the patient attains optimal physical condition and the cavity is clean. This ensures the greatest chance for success [4]. It should be understood that adequate drainage of the pleural cavity is “sine quo non” in the effective management of BPFs and alone carries the possibility of fistula closure as in our case. Instillation of antibiotic solution and chest closure without addressing the infection is always headed to fail.

Identification of the BPFs should be followed by assimilation of other pertinent information. Location, either central or peripheral, and Size of the fistula should be identified along with its relationship to the bronchial tree and other adjacent structures. This information, coupled with the identification of the apparent cause of BPF, can help physicians come up with an apt treatment plan. The consensus seems to be that smaller fistulas should be corrected conservatively and/or endoscopically if need be, and larger fistulas should be treated with surgical interventions [4,6-8]. BPFs more than 8 mm are not suitable for endoscopic management, while those 1 mm in size have the highest success rate. In terms of Location, distal small BPFs have better outcomes with bronchoscopic interventions, while large or central BPFs are best managed with surgery or stent placement. Surgical measures like video-assisted thoracoscopic surgery (VATS) decortication or open decortication have been shown to have lower readmission risk and mortality compared to chest tubes with or without intrapleural fibrinolysis [3].

In high-risk surgical patients, endoscopic procedures, including the use of sealants or placement of endobronchial valves, may serve as a temporary measure until clinical optimization is achieved. In other patients deemed nonsurgical candidates, the procedure may be the only option [12].

Bronchoscopic endobronchial valve placement is minimally invasive, with a high success rate for management of BPFs. Especially in COVID-19 cases, where there is significant perioperative risk, EBV deployment can be a useful tool in our arsenal. However, there is a big question regarding the safety of providers, given the COVID-19 transmission. Studies show the risk appears to be low when recommended precautions like appropriate use of PPEs and use of neuromuscular blockers to prevent cough are followed [11,13].

The purpose of this literature review is to outline the incidence of BPFs with empyema in the setting of COVID-19, which has a dismal prognosis. Awareness about BPF in the setting of COVID-19 can guide providers in the appropriate diagnosis and management of this condition. While a definite consensus is yet to be established in the medical community, available literature advocates management of BPFs with effective drainage with the use of extracorporeal membrane oxygenation (ECMO), mechanical ventilation in cases of severe hypoxia, and definitive management in the form of endoscopic or surgical interventions. General intensive care, including nutrition and physical rehabilitation, should be initiated as soon as possible [14]. None of the prevention methods guarantees the complete elimination of BPF formation, and there is no universal method of BPF treatment. In such a situation, even greater individualization of treatment is necessary [15].

## Conclusions

Polymicrobial empyema with bronchopleural fistula in the setting of COVID-19 represents a rare and complex clinical challenge. This case illustrates that early recognition, prompt drainage, and tailored antibiotic therapy can lead to successful outcomes, even in the presence of a BPF, without requiring surgical intervention. It emphasizes the importance of a multidisciplinary, conservative approach in managing select thoracic complications of COVID-19, potentially avoiding more invasive procedures in appropriately selected patients. As we learn more about COVID-19 complications, physician needs to watch for unusual cases like this. This report suggests that, with the right care and teamwork, less invasive treatments can work well for selected patients with complex chest infections.
